# A retrospective study of individualized endovascular treatment for symptomatic intracranial atherosclerotic stenosis in patients with ischemic stroke/transient ischemic attack

**DOI:** 10.3389/fneur.2022.1057935

**Published:** 2022-11-30

**Authors:** Lun-Lin Mao, Ai-Jin Ma, Zhi-Qing Liu, Jin Zhang, Yuan-Feng Xu, Wen-Ya Chen, Yong-Jun Cao

**Affiliations:** ^1^Department of Neurology, The Second Affiliated Hospital of Soochow University, Suzhou, China; ^2^Department of Neurology, Wujin Hospital Affiliated to Jiangsu University, The Wujin Clinical College of Xuzhou Medical University, Changzhou, China

**Keywords:** ischemic stroke, endovascular treatment, balloon dilation angioplasty, stent implantation, intracranial atherosclerotic stenosis

## Abstract

**Background:**

Endovascular treatment (EVT) is one of the effective treatment procedure for the symptomatic intracranial atherosclerotic stenosis (sICAS).

**Aim and methods:**

We evaluated the efficacy and safety of individualized endovascular treatment for sICAS patients. Clinical and imaging follow-ups were carried out to collect the data of 29 sICAS patients after 6 months of individualized endovascular treatment. Different treatment strategies are selected based on arterial access and lesion morphology of patients. If standard surgical path, narrow artery straight, stenosis length ≤10 mm, then the appropriate specifications of balloon-mounted stent (BMS) treatment. the surgical path is tortuous, the narrow artery is curved, the angle is apparent, the diameter of the near and far ends is significantly different, or the length of the stenosis is >10 mm, self-expanding stent (SES) with appropriate specifications is selected for treatment. If the narrowed artery is hyper flexed and the surgeon deems stenting inappropriate, balloon dilation angioplasty (BDA) treatment is chosen.

**Results and conclusion:**

31 lesions of 29 sICAS patients received endovascular treatment. The median age was 61 years (IQR 54–69 years). The median preoperative stenosis was 90% (IQR 80–95%), and the mean stenosis length was (8.10 ± 3.27) mm. The most commonly used surgical procedure was Balloon-Mounted Stent (BMS) in 19 cases (65.52%), Self-expanding Stent (SES) in seven cases (24.14%), Balloon Dilation Angioplasty (BDA) in three cases (10.34%). (11.86 + 1.46 mm) was greater than that in the BMS group (6.14 + 1.59 mm) (*P* < 0.001). The median stenosis was 90% (IQR 80–92.5%) in the BMS group, lower than 99% (IQR 95–100%) in the SES group (*P* < 0.001). The median post-operative residual stenosis was 20% (IQR 15–25%), significantly improved compared with preoperative (*P* < 0.001). The success rate of the surgical technique was 93.10% (27/29). One patient (3.45%) had IS recurrence within 48 h after surgery, and the restenosis rate within 6 months after surgery was 6.90% (2/29). No patient died or had recurrent IS. Our data demonstrated that individualized endovascular treatment method could be potentially significant and safe for sICAS patients. This study will provide an important reference for the endovascular treatment of sICAD.

## Introduction

After ischemic heart disease, stroke is the second most significant cause of mortality globally (1), and ischemic stroke (IS) accounts for 87% of all instances (2). Intracranial atherosclerotic stenosis (ICAS) is one of the leading causes of IS, accounting for about 30–50% of IS in Asia (3).

To prevent recurrent transient ischemic attacks (TIAs) and IS, medical management, which includes antiplatelet treatment, comprehensive cardiovascular risk factor control, and lifestyle management, is still advised as the first-line medical treatment for ICAS. Despite extensive medical care, patients with high-grade (70–99%) symptomatic ICAS continued to have a substantial risk of recurrent TIA and IS. This particular group of patients was regarded as refractory to aggressive medical therapy (4). A study called “Stenting and Aggressive Medical Management for Preventing Recurrent Stroke in Intracranial Stenosis trial (SAMMPRIS trial)” reported that the risk of stroke or mortality within 1 year in symptomatic ICAS patients with more than 70% of stenosis was as high as 12.6% (5). Moreover, every patient in the medical arm received a lifestyle coach, which is unlikely to be offered by general healthcare systems, particularly in low- or middle-income nations (6). Therefore, to avoid recurrent TIA or IS in patients with a high degree of ICAS, endovascular therapy, such as balloon angioplasty alone, balloon-mounted stent implantation, or self-expandable stent placement, could be considered an alternative treatment option.

Although the SAMMPRIS and VISSIT (the Vitesse Intracranial Stent Study for Ischemic Stroke Therapy) trials suggested that aggressive medical management was preferable to stent therapy (5, 7), some prospective and retrospective studies from Asia and Europe reported encouraging outcomes for endovascular treatment (8–13). This study aims to retrospectively analyze the prognosis of individualized endovascular treatment for 6 months and evaluate the efficacy and safety of individualized endovascular therapy in patients with ICAS.

## Methods

### Patients

This study was conducted at the Advanced Stroke Center of Wujin Hospital, affiliated with Jiangsu University, as a single-center retrospective cohort study. Retrospectively collected 29 patients with severe ICAS (70–100%) confirmed by DSA from January 2019 to June 2021 who received individualized endovascular therapy, including the intracranial (C5–C7) segment of the internal carotid artery, the middle cerebral artery level (M1) segment, and the vertebral intradural (V4) segment. All patients sign an informed consent form before surgery.

The inclusion criteria are developed by the executive committee as follows: (A) age >18 years; (B) TIA or IS due to severe intracranial artery stenosis (≥70%); (C) poor compensation of cerebral collateral circulation, cerebral CT perfusion suggests hypoperfusion in the responsible vascular supply area; (D) high-resolution magnetic resonance vascular wall imaging, which identifies the cause of vascular stenosis as atherosclerosis based on the thickening of the vascular wall and the way in which it is reinforced, and at least one risk factor for atherosclerosis (hypertension, diabetes, hyperlipidemia, hyperhomocysteinemia, and smoking) (10, 14, 15). (E) Individualized endovascular treatment strategies are selected based on arterial access and lesion morphology of patients.

Exclusion criteria include:

(A) The acute phase of IS (≤24 h);
(B) Non-atherosclerotic stenosis of intracranial arteries includes arterial dissection, vasculitis, and muscle fiber dysplasia;
(C) Concomitant extracranial artery tandem stenosis, intracranial tumor, cerebral aneurysm, or arteriovenous malformations;
(D) Contraindications to the use of aspirin, clopidogrel, heparin, tirofiban, contrast agents, metals, etc;
(E) Life expectancy < 1 year because of other diseases, such as cancer.
(F) Asymptomatic stenosis and untraceable patients;
(G) Have received endovascular treatment before the responsible lesion;
(H) Other situations where endovascular treatment is not suitable.

### Device selection and surgical procedure

A Balloon-Mounted Stent (MicroPort Medical, Shanghai, China), Self-expanding Stent (Wingspan, Boston Scientific), and Balloon Dilation Angioplasty (BDA) were chosen according to the characteristics of the lesions and the experience of the surgeon. In general, the self-expanding stent was chosen in patients with tortuous arterial access, a Mori C lesion, or a lesion with a significant mismatch in the diameter between the proximal and distal segments, while the Apollo balloon-mounted stent was chosen in patients with smooth arterial access and a Mori A lesion. If the narrowed artery is hyper-flexed and the surgeon deems stenting inappropriate, the BDA treatment could be selected.

All patients in our study received aspirin (100 mg/day) and clopidogrel (75 mg/day) in dual antiplatelet therapy at least 5 days before the surgery while receiving strict glycemic, smoking control, and lipid-lowering (atorvastatin calcium tablets 20–40 mg/day) and standardized blood pressure management. Depending on the patient's condition and the operator's experience, either general anesthesia or local anesthesia was selected. The surgical procedure was mainly carried out in the following steps. After local infiltration anesthesia, as high as the vascular tortuosity permitted, the 6F or 8F guiding catheter was pushed into the internal carotid artery. A 0.014-inch micro-guidewire was used to traverse intracranial artery stenosis using road mapping, and an angioplasty catheter was then injected over the stenosis. Recanalization was deemed effective if there was less than 50% residual stenosis following stent implantation. A thorough neurological examination and head CT were performed after the surgery to rule out an IS or potential hemorrhage.

### Perioperative management

All patients received aspirin (100 mg/day) and clopidogrel (75 mg/day) dual antiplatelet therapy for at least 5 days before surgery. They also received strict glycemic, smoking control, and lipid-lowering (atorvastatin calcium tablets 20–40 mg/day) and maintained their standard blood pressure. The patient needs to be maintained for at least 6 months after surgery with dual antiplatelet therapy, after which aspirin or clopidogrel alone is continued daily. Thromboelastographic (TEG) is carried out to determine whether aspirin resistance (arachidonic acid inhibition rate <50%) or clopidogrel resistance (adenosine biphosphate inhibition rate <30%) (16), the ticagrelor or cilostazol can be selected as an alternative. The following objectives were executed with aggressive medical therapy: systolic blood pressure 140 mm Hg, low-density lipoprotein 70 mg/dl (1.81 mmol/L) or a 50% drop, quitting smoking, lifestyle changes for obesity, and sedentary behavior. Rehabilitation is recommended for patients with functional disabilities and long-term management of risk factors such as hypertension, diabetes, hypercholesterolemia, and hyperhomocysteinemia.

### Clinical and imaging follow-up

This retrospective study's clinical outcomes included mortality or stroke event, ipsilateral stroke event, and TIA recurrence within 6 months of endovascular therapy. A successful endovascular operation was defined as having less than 50% of the initial stenosis still present and having successfully implanted a stent over the stenosis lesion. In-stent thrombosis, vasospasm, arterial dissection, arterial perforation, pseudoaneurysm, groin hematoma, and cerebral bleeding are procedure-related problems and hemorrhages brought on by increased cerebral blood flow.

A DSA examination is included in the follow-up plans for the endovascular operation. All patients were followed up for 6 months. But, after surgery, patients received clinical and imaging follow-up and the median clinical follow-up time was about 6 months (±6 months) for MRA exam/CTA/CTP (CT perfusion) scans. As determined by imaging analysis, the presence of greater than 50% stenosis was considered a recurrence of ICAS. At least two experienced neurosurgeons reviewed the immediate clinical and follow-up results. The follow-up period's ICAS recurrence and procedural problems were promptly managed.

### Statistical analysis

SPSS 19.0 statistical software was used to process the data statistically. The Kolmogorov-Smirnov test first evaluated the numerical variable data, the measurement data in line with the normal distribution were expressed as mean ± standard deviation (±SD), the mean comparison between the two groups was *t*-tested by two independent samples, and the comparison of vascular stenosis rate before and after treatment of patients was compared with paired *t*-test. The abnormal distribution of numerical variables is expressed in median (M) and quartile spacing (Q), and the comparison between the two groups is tested by two independent samples Mann-Whitney *U*. The categorical variable data are expressed in frequency and percentage, and the difference between *P* < 0.05 is statistically significant.

## Results

### Baseline characteristics

A total of 29 patients had pre-operative events, with a median age of 61 years (IQR 54–69 years), with 19 males (65.25%) accounting for the majority. In 26 cases (89.66%), hypertension is the most common risk factor for cerebrovascular disease. At admission, the median NIHSS score was 1 (IQR 0.75–2). The median time from the onset of an IS event to endovascular therapy was 21 days (IQR 15–27 days). Most lesions are located in the anterior circulating middle cerebral artery (41.38%, 12/29). Two cases of the V4 segment of the left vertebral artery had both proximal and fusion segment severe tandem stenosis, and both were treated with BMS. Characteristics of stenosis lesions in all patients: median degree of stenosis (WASID) before interventional therapy was 90% (IQR 80–95%), and the average length of lesions was (8.10 + 3.27) mm. The post-operative median residual stenosis degree is 20% (IQR 15–25%). The most commonly used surgical methods were 19 cases of BMS (65.52%), 7 cases of SES (24.14%), and 3 cases (10.34%) of patients (10.34%) were unsuitable for stent placement due to excessive tortuous lesions. BDA treatment was finally carried out to avoid difficulty in stent placement and poor stent formation and reduce intraoperative complications. The mean length of lesions in the SES group (11.86 + 1.46 mm) was greater than that in the BMS group (6.14 + 1.59 mm) (*P* < 0.001). The degree of stenosis in the BMS group was 90% (IQR 80–92.5%) compared with 99% (IQR 95–100%) (*P* < 0.001) in the SES group, and the difference was significant. The baseline characteristics are shown in Table 1.

**Table 1:** Demographic features, intracranial artery stenosis characteristics, and surgical modalities.

**Demographic characteristics**	***N*** **= 29**
Age (median, intraluminal spacing)	61 (53.5–68.5)
Male	19 (65.52)
Hypertension	26 (89.66)
Hyperlipidemia	11 (37.93)
Type 2 diabetes	12 (41.38)
Obesity	13 (44.83)
Hyperhomocysteinemia	10 (34.48)
Atrial fibrillation	2 (6.89)
Coronary heart disease	2 (6.89)
Smoking	12 (41.38)
History of stroke	2 (6.89)
Admission NIHSS score (median, intraluminal spacing)	1 (0.75–2)
Onset to endovascular treatment time (d) (median, intraluminal spacing)	21 (15–27)
**Features of intracranial artery stenosis**	
Percentage of preoperative mean stenosis (WASID) (median, intraluminal spacing)	90 (80–95)
The average length of stenosis is (mm)	8.10 ± 3.27
Left vertebral artery in the medial segment	6 (20.69)
The right vertebral artery in the lindural segment	4 (13.79)
Basal arteries	3 (10.34)
Left intracranial segment of the internal carotid artery	4 (13.79)
Intracranial segment of the right internal carotid artery	2 (6.90)
Left middle cerebral artery	6 (20.69)
Right middle cerebral artery	6 (20.69)
**Surgical modalities**	
Simple balloon dilation angioplasty (BDA)	3 (10.34)
Self-expanding stenting (SES)	7 (24.14)
Balloon dilated stenting (BMS)	19 (65.52)

### Post-operative perioperative outcomes of endovascular therapy

**Figure 1:**
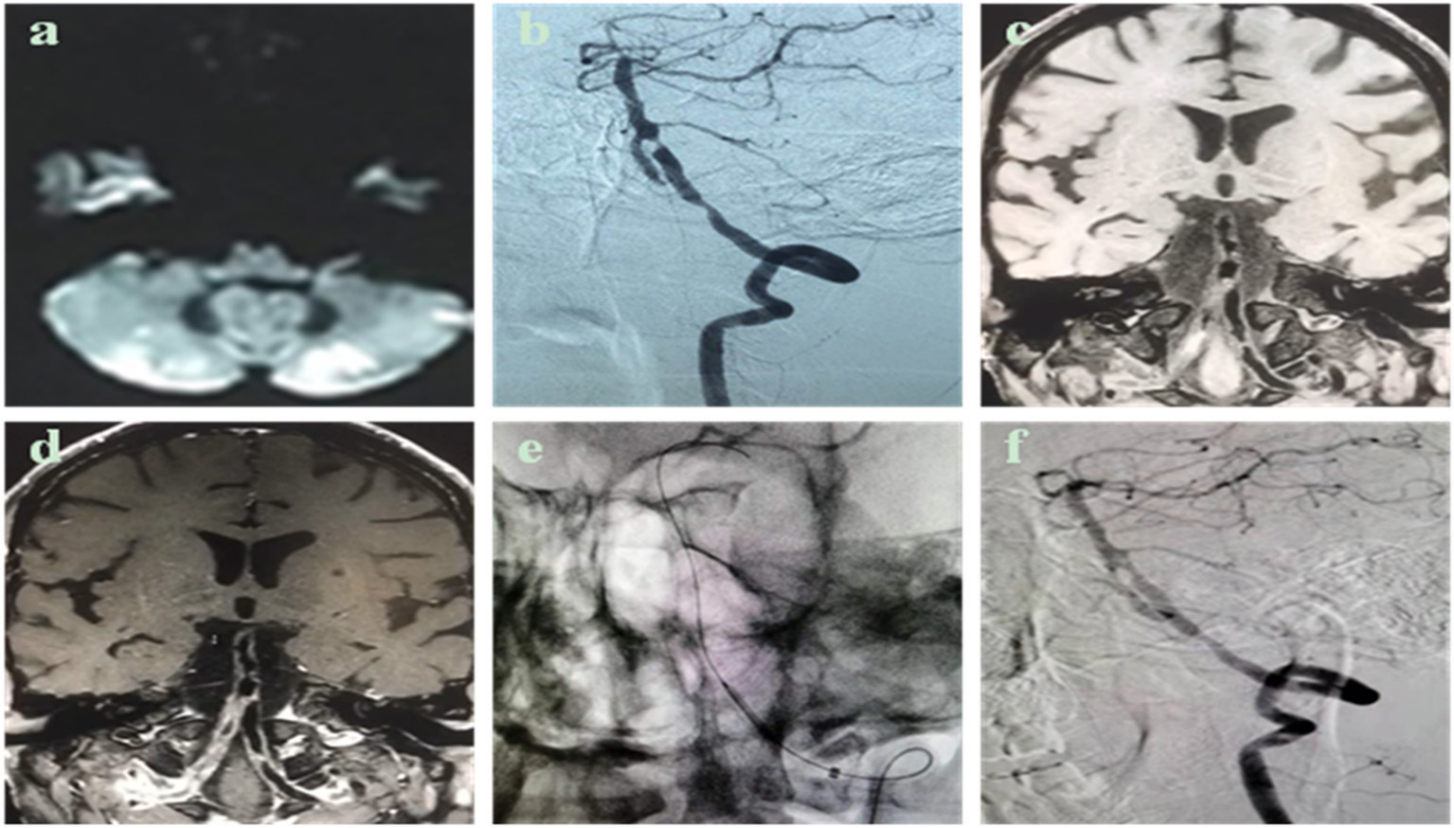
Case description: Male, 50 years old, admitted to the hospital for 1 month due to dizziness and walking instability, who was treated in an external hospital, and the head magnetic resonance imaging showed multiple acute cerebral infarctions in both cerebellar hemispheres. He has a history of hypertension and cerebral infarction and has received antiplatelet and lipid-lowering therapy. Have a history of smoking. Cerebral angiography shows occlusion of the medial segment (V4) of the right vertebral artery and severe tandem stenosis of the V4 segment of the left vertebral artery. After admission, high-resolution magnetic resonance imaging of the intracranial vascular wall was completed, suggesting that the V4 segment of the left vertebral artery was a narrowing caused by atherosclerotic plaque. **(a)** Cranial MRI DWI sequence shows multiple points and flaky hyperinflation of the cerebellar hemisphere on both sides; **(b)** cerebral angiography sees occlusion of the V4 segment of the right vertebral artery, 70% of the proximal stenosis of the V4 segment of the left vertebral artery, and 90% of the proximal fusion segment of the distal, proximal fusion segment; **(c,d)** high-resolution MRI vascular wall imaging shows an eccentricity of the V4 segment of the left vertebral artery, focal thickening, local bleeding in the plaque, plaque strengthening; **(e,f)** The left vertebral artery V4 segment tandem stenosis lesion is pre-dilated sequentially with a balloon of 2.0 mm × 15 mm. The proximal stenosis was placed with a 3 mm × 13 mm spherical expansion stent, and the distal stenosis was placed with a 2.5 mm × 10 mm spherical expansion stent, and the post-operative angiography showed that the left vertebral artery V4 segment tandem stenosis improved, and the residual stenosis ≤30%.

The degree of post-operative median residual stenosis (WASID) is 20% (IQR 15–25%) (Table 2). A patient with left V4 proximal and fusion segment stenosis and chronic occlusion of the right vertebral artery (Figure 1), whose posterior circulation mainly relied on the left vertebral artery to supply blood. There was brief dizziness, nausea, and vomiting during balloon expansion, which was an inevitable cerebral ischemia manifestation during the operation. A post-operative review of the skull MRI ruled out new cerebral infarction. There were two cases of post-operative cerebral angiography (one case of left internal carotid artery intracranial segment and one case of right middle cerebral artery M1 segment BDA alone) with residual stenosis >30%. Still, the distal blood flow was smooth, and the forward blood flow reached mTICI grade 3. The overall success rate of vascular reconstitution was 93.10% (*n* = 27). The rate of post-operative stenosis in endovascular therapy decreased from a median of 90% (IQR 80–95%) to 20% (IQR 15–25%) (*P* < 0.001), and the difference was statistically significant.

**Table 2:** Overall prognosis.

**Prognostic indicators**	***N*** **= 29**
mRS score (median, interquartile interval)	1 (0–1)
Percentage of residual stenosis after surgery (WASID) (median, interstitial spacing)	20 (15–25)
Surgical technique (success rate)	27 (93.10)
Perioperative complications and death	1 (3.45)
Recurrent IS and TIA during follow-up	0
Vascular restenosis rate	2 (6.90)
Mortality during follow-up	0 (0)

Skull CTA examination within 48 h after surgery found that 15 cases of no residual stenosis, 13 cases of residual stenosis were ≤50%, 1 case had stent thrombosis, vascular occlusion (the patient was M1 segment stenosis of the right middle cerebral artery, no residual stenosis after BMS surgery) and symptomatic IS, and then the emergency department used BMS remedial retreatment, occlusive blood vessels were successfully re-passed, residual 20% stenosis, a small amount of thrombosis escaped at the distal end, the patient was finally recovered and discharged, and the mRS was 1 point when discharged. Except for one patient (3.45%) who developed IS within 48 h after surgery, the rest of the patients did not have any stroke and TIA events during the perioperative period, and there were no deaths. Thromboelastic testing excludes resistance to aspirin and clopidogrel 1.

### Six-month follow-up results

All patients received outpatient or telephone follow-up after surgery, and there were no cases of loss of visit. There were no recurrent IS and TIA events during the follow-up period, no deaths, and an excellent clinical prognosis (median mRS 1). The CTA examination of the skull at the follow-up at the 6th month after surgery found that 15 patients had no signs of stenosis, 12 cases of stenosis ≤50%, and 2 cases (6.9%) of the patients had asymptomatic restenosis, of which 1 case was treated with BDA for left internal carotid artery intracranial stenosis, and 1 case was treated with SES treatment of M1 segment stenosis of the right middle cerebral artery, both of which were hypertension and type 2 diabetes mellitus (Table 2).

## Discussion

Currently, there are limited data on the study of individualized endovascular therapy for sICAS. This study retrospectively analyzed the effectiveness and safety of individualized endovascular treatment in sICAS. The results showed that the incidence of intracranial perioperative complications was 3.45%, the technical success rate was 93.1%, the median stenosis was improved from 90 to 20%, and there was no recurrent IS, TIA, and death within 6 months of follow-up, and the restenosis rate was 6.9%. In the SAMMPRIS study, the complication rate in the ICAS treatment group with SES for 30 days was 14.7%, and the recurrence of IS and death after 1 year of follow-up reached 20% (5). In the VISAIT study, the 30-day complication rate of ICAS was 24.1% in the BMS treatment group, and the 1-year incidence of IS and TIA was 36.2% (7). Therefore, double antiplatelet joint risk factor control is still used as a first-line treatment for patients with sICAS. However, the 1 year IS recurrence rate in the drug treatment group in SAMMPRIS and VISSIT studies is also higher, 12.6 and 15.1% (5, 7), respectively, indicating that aggressive drug therapy is insufficient to achieve the expected preventive effect. In addition, the safety and efficacy of endovascular treatment for ICAS have been questioned due to the high perioperative complication rates and high IS recurrence rates during follow-up studies studied by SAMMPRIS and VISSIT. Therefore, endovascular treatment in patients with severe stenosis (≥70%) remains challenging.

In addition, it is important to note that studies have shown that most early IS recurrences occur in the first few days after the initial ischemic event (17, 18). The median time of patients included in the SAMMPRIS and VISSIT studies was 7th and 9th day, respectively. Recurrence of ischemic events may have occurred before inception, which helped to observe lower stroke and TIA incidences in the drug group. Patients with early stroke recurrence may also be the ones who benefit most from early endovascular intervention. Patients with sICAS have also been reported to receive endovascular therapy within 24 h of symptoms and have a higher risk of recurrence of perioperative stroke (19). To mitigate this effect, the WEAVE study included patients more than 7 days after an ischemic event, resulting in low stroke and mortality (2.6%) in the perioperative period (≤72 h) after stenting. The median time from onset to endovascular treatment in this study was 21 days, all of which avoided the highest risk period for stroke recurrence, which may help reduce the risk of intraoperative and post-operative complications. Therefore, the best period for endovascular therapy for ICAS is controversial.

Because a thin tube wall and easy rupture, more perforation and easy occlusion, and the complex characterize the stenosis of the intracranial artery path curvature makes it difficult for stents to be in place during endovascular treatment. Therefore, the safety of the endovascular treatment is an urgent need to be solved in the clinical treatment of ICAS. Neuro-surgeons have come to realize that the individual selection of endovascular therapeutic materials appropriate to the characteristics of ICAS lesions has an important impact on perioperative complications (20, 21). Both the SAMMPRIS and VISSIT studies were questioned for restricting the use of a single stent to treat different types of ICAS. By drawing on the experience of the SAMMPRIS trial and prospectively evaluating the feasibility and safety of individualized endovascular therapy in ICAS patients according to the vascular pathway and lesion morphology, the results showed that the technical success rate reached at 96.3%. The sever stroke, myocardial infarction, or mortality rate of 30 days after surgery was 4.4%, which was comparable to the results of this study. However, the medium- to the long-term efficacy of individualized endovascular therapy for ICAS has not been explored. In addition, patients with symptoms of intracranial hypoperfusion and poor collateral circulation are most likely to benefit from endovascular treatment because of inadequate drug therapy. The experience of neuro-interventional physicians also plays a key role in the success rate of endovascular treatment (22). The patients selected for this study were all operated on by experienced neuro-surgeons, which provided a guarantee to improve the success rate of surgery and reduce the risk of complications.

It has been reported that balloon dilation angioplasty alone has similar perioperative complication rates compared with stenting angioplasty in the treatment of ICAS (23). A long-term complication of endovascular therapy with ICAS is secondary to endometrial hyperplasia due to balloon dilation or mechanical micro-injury during stenting. If lumen is stenosis≥, 50% may re-trigger a cerebral ischemic event (24). Therefore, it is crucial to prevent such lesions. Studies have shown that treating sICAS with drug-coated balloons with antiproliferative drugs is safe and feasible and can reduce intracranial complications and restenosis rates (25–27). However, a recent retrospective study of drug-coated balloon dilation angioplasty for sICAS alone showed a 6% complication rate at 30 days post-operatively, and an asymptomatic IS recurrence rate of 12% at a median follow-up of 9 months (28), both higher than in this study. The patients in this study all had a history of stroke, including male proportion, patient age, hypertension, diabetes, hyperlipidemia, IS events to endovascular treatment time. However, one study showed that 14.4% of patients still had ingratiatory restenosis (stenosis of more than 70%) within 1 year (29). In addition, a previous study showed that the average follow-up rate of ICAS after Enterprise stent treatment was 24.7% (stenosis exceeded 50%), which was higher than the restenosis rate (6.9%) (Stenosis more than 50%) at 6 months follow-up in this study (30). Therefore, this study suggests that choosing an individualized endovascular treatment for the ICAS patients may be more beneficial in reducing perioperative complications, the risk of long-term IS recurrence and vascular restenosis.

## Limitations

This study has some limitations. This study was a single-center, non-randomized controlled retrospective study in which only Chinese in the region was not representative, and the number of patients included was small, making it impossible to compare the prognosis and related factors of the three surgical modalities.

## Conclusion

Our study showed that individualized endovascular treatment of sICAS patients might be effective and safe, potentially improve the technical success rate, and reduce perioperative complications and the recurrence rate of IS within 6 months. However, the results of our study still need to be further confirmed by multicenter large-sample randomized controlled studies.

## Data availability statement

The original contributions presented in the study are included in the article/supplementary material, further inquiries can be directed to the corresponding author/s.

## Ethics statement

The studies involving human participants were reviewed and approved by the Ethics Committee at Wujin Hospital, Affiliated with Jiangsu University. The patients/participants provided their written informed consent to participate in this study.

## Author contributions

Y-JC was responsible for the conception and design of the work, revising it critically for important intellectual content and final approval of the version to be published. W-YC made substantial contributions to the conception and design of the work and revising it critically for important intellectual content. L-LM was responsible for the conception and design of the work as well acquisition, analysis, and interpretation of data and writing the manuscript. A-JM, Z-QL, JZ, and Y-FX collected and supplied the clinical data. All authors contributed to the article and approved the submitted version.

## Funding

The Science and Technology Project (Social Development) of Wujin District (Nos. WS202019 and WS202112) and the Science and Technology Development Foundation of the affiliate hospitals of Xuzhou Medical University (No. XYFY2020042) supported this work.

## Conflict of interest

The authors declare that the research was conducted in the absence of any commercial or financial relationships that could be construed as a potential conflict of interest.

## Publisher's note

All claims expressed in this article are solely those of the authors and do not necessarily represent those of their affiliated organizations, or those of the publisher, the editors and the reviewers. Any product that may be evaluated in this article, or claim that may be made by its manufacturer, is not guaranteed or endorsed by the publisher.
